# Recurrent amebic liver abscesses despite metronidazole treatment: A rare case report

**DOI:** 10.1002/ccr3.8138

**Published:** 2023-11-02

**Authors:** Sasan D. Noveir, Anh Hoang, Katherine Li, John C. Lam, Khushboo Akkad

**Affiliations:** ^1^ David Geffen School of Medicine Los Angeles California USA; ^2^ Department of Emergency Medicine University of California Los Angeles Los Angeles California USA; ^3^ Department of Medicine, Division of Infectious Diseases University of California Los Angeles Los Angeles California USA; ^4^ Department of Medicine, Division of Hospital Medicine University of California Los Angeles Los Angeles California USA

**Keywords:** amebiasis, *Entamoeba histolytica*, liver abscess, amebic, parasitic diseases, paromomycin

## Abstract

Amebic liver abscesses should be considered in adult males with a liver abscess and a history of travel to endemic areas. Effective treatment includes metronidazole, followed by paromomycin.

## Graphical abstract

CT scan shows a percutaneous drain in the patient’s fourth liver abscess (blue arrow) and the occurrence of a new, fifth liver abscess (red arrow).

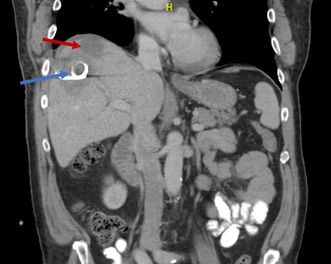

## 1 BACKGROUND

Amebiasis is an intestinal infection with fecal‐oral transmission caused by *Entamoeba histolytica*, a parasitic amoeba. Other Entameoba species, including *Entamoeba dispar, Entamoeba moshkovskii, Entamoeba polecki, Entamoeba coli*, and *Entamoeba hartmanni*, are not known to be pathogenic and likely represent intestinal colonizers. While amebiasis is asymptomatic in 80% of cases, it can present with abdominal pain, diarrhea, or constitutional symptoms. Extraintestinal manifestations of amebiasis can occur from invasive infection, with amebic liver abscess (ALA) being the most common complication.^1^ While rates of asymptomatic amebiasis are equal in both sexes, ALAs are more prevalent in men.^2^ Risk factors for infection include travel to endemic areas with poor sanitary conditions, usually from contaminated food or water. Clinical manifestation of the disease typically occurs 8–20 weeks after exposure but can occur decades after initial exposure.^3^ The length of stay in endemic areas is not associated with an increased risk of ALA, underscoring the importance of a complete travel history.^3^

Individuals with ALA often present acutely with fever, right upper quadrant pain, and, occasionally, a cough. Subacute anorexia and weight loss are also reported, with diarrhea notably absent.^1^, ^4^ Radiographically, ALAs are variably‐sized solitary collections in the right hepatic lobe, resulting from the preferential portal circulatory system of the right colon.^5^ Common complications of ALA include transudative pleural effusions or abscess rupture leading to pleuropulmonary amoebiasis, with a possible misdiagnosis of bacterial pneumonia.^5^ Rarely, the mechanical compression and inflammation of an ALA cause hepatic vein and inferior vena cava thrombosis.^6^

The diagnosis of ALA is challenging and relies on imaging and serological *E. histolytica* testing in the appropriate clinical context. Polymerase chain reaction (PCR) of liver aspirate may be helpful, as stool PCR can be negative without concurrent amebic colitis.^1^ Treatment of ALA includes a 10‐day regimen of metronidazole 750 mg orally three times a day, followed by an intraluminal amebicide such as paromomycin 10 mg/kg orally three times a day. Decompression is not required but may be helpful to confirm the diagnosis or if there is a concern for rupture or a lack of response to medical therapy.^7^

## 2 CASE SUMMARY

**FIGURE 1:**
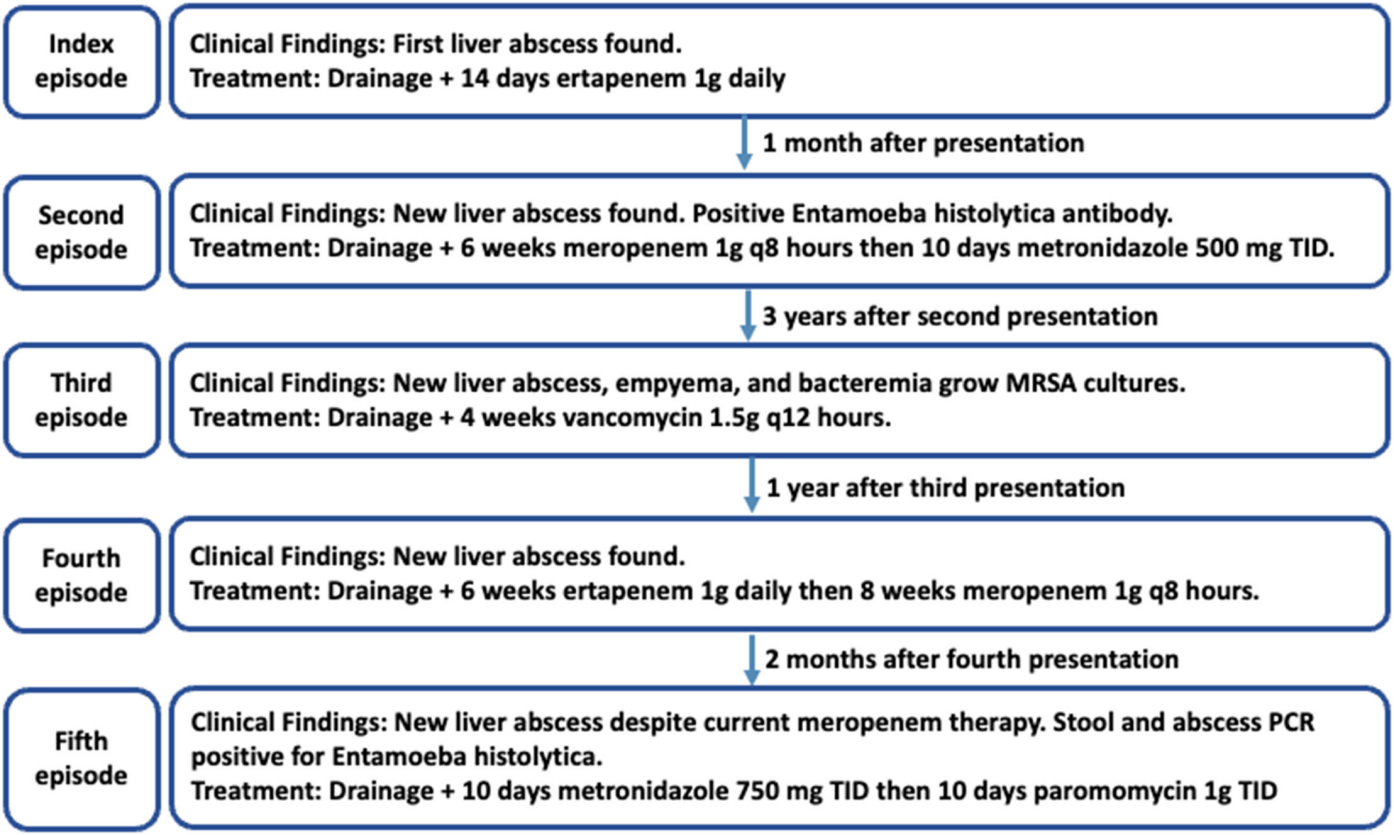
Timeline of the patient's clinical course with respect to amebic liver abscess recurrences.

A previously healthy 66‐year‐old man was admitted to the hospital for a workup of recurrent culture‐negative liver abscesses refractory to meropenem therapy. He presented with his fifth occurrence of a hepatic abscess over the past 5 years (Figure 1 **)**. The patient lives independently in urban Nevada, with no exposure to animals or engagement in outdoor activities. There is no history of housing instability or incarceration. His sexual history included insertive vaginal intercourse with barrier protection with female partners. His only travel outside the United States was an uneventful 5‐day trip to Mexico 30 years ago. Several months before his first liver abscess, he used subcutaneous synthetic analogues of growth hormone‐releasing hormone (GHRH) for muscle building. His index presentation was a subacute history of fever, chills, and fatigue, where he was found to have a liver abscess and treated with 14 days of ertapenem.

One month following ertapenem therapy, he presented with similar constitutional symptoms and was found to have recurrent hepatic abscess formation. He was treated with 6 weeks of meropenem. Serum *E. histolytica* antibody was positive, and he completed a 10‐day course of metronidazole 500 mg orally thrice daily. It is unclear whether he completed paromomycin after that.

Three years after his second episode, he was hospitalized with fever, chills, fatigue, and severe dyspnea. He was found to have methicillin‐resistant *Staphylococcus aureus* (MRSA) bacteremia and empyema. Cross‐sectional imaging revealed the recurrence of a liver abscess, which was aspirated with MRSA. Following percutaneous drainage, a 4‐week course of intravenous vancomycin was administered with radiographic improvement. Studies for *Entamoeba* were not sent as it was believed his liver abscess was pyogenic from MRSA.

**FIGURE 2:**
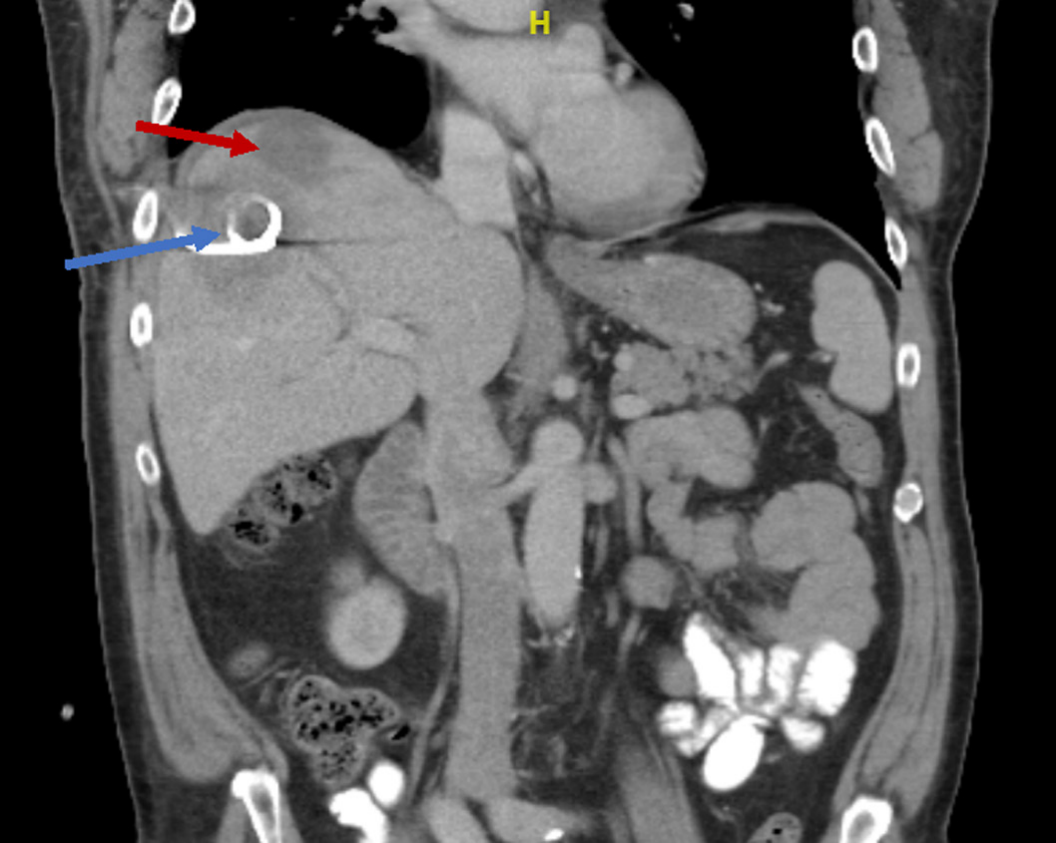
CT abdomen and pelvis with contrast from the patient's fifth episode of recurrent liver abscess. This shows a large abscess (red arrow) within the liver dome, measuring 8.2 cm. There is a heterogeneous abnormal signal within the right hepatic lobe inferior to the liver dome abscess, corresponding to the previously drained abscess cavity; the percutaneous drainage catheter is noted within this region (blue arrow).

One year later, he was found to have a recurrence of culture‐negative hepatic collection. Despite completing ertapenem for 6 weeks and current treatment of meropenem for 3 weeks with patent percutaneous drains, serial cross‐sectional imaging demonstrated a previously drained right lobe abscess cavity, a new right lobe abscess, and a thrombus of the anterior segment right hepatic vein (Figure 2).

The patient was admitted to the hospital for evaluation of this new liver abscess with an adjacent thrombus. The patient noted 60 pounds of weight loss throughout the last 5 years but had no abdominal pain, diarrhea, bloody stools, nausea, or vomiting. The physical examination was non‐contributory. Laboratory investigations were only significant for normocytic anemia and mildly elevated alkaline phosphatase. The aspiration of the enlarging hepatic collection was negative for bacteria, acid‐fast bacilli, and fungus. Doxycycline was added to meropenem for acellular coverage. Esophagogastroduodenoscopy, colonoscopy, and magnetic resonance enterography did not demonstrate evidence of malignancy or inflammatory bowel disease. The PCR of the stool and the liver abscess were positive for *E. histolytica*. Antibacterials were discontinued, and the patient was discharged with a 10‐day course of metronidazole 750 mg orally thrice daily, followed by 10 days of paromomycin 1000 mg orally thrice daily. Repeat stool PCR 4 weeks after treatment was negative for stool ova and parasites. Stool PCR to screen for asymptomatic carriers among the patient's family members all yielded negative results.

## 3 DISCUSSION

There are several possible considerations for the recurrent nature of the patient's liver abscesses. The initial treatment with metronidazole at 500 mg thrice daily may have been subtherapeutic, as current guidelines recommend dosing at 750 mg thrice daily. One study in India found a recurrence rate of 9% within 2 years among patients not treated with a luminal agent.^8^ Another study showed a 72% prevalence of asymptomatic luminal colonization of *E. histolytica* at the initial presentation of ALA. Of these patients with concomitant intestinal infection, treatment with metronidazole was insufficient to eradicate intestinal infection in greater than 50% of patients, despite resolution of the liver abscess.^9^ Additionally, metronidazole therapy without a subsequent luminal agent is ineffective in addressing asymptomatic colonization.^10^, ^11^, ^12^ *E. histolytica* can develop metronidazole resistance in vitro, but rates of clinical resistance are unclear.^13^ Another possibility considered for the patient's recurrence was close contact with asymptomatic carriers, such as his family. However, the patient's family tested negative for the stool *E. histolytica* antigen.

It is uncertain when the patient was initially infected with *E. histolytica*. While his use of exogenous GHRH preceded his first liver abscess, it remains unclear whether GHRH increased his susceptibility to ALA recurrence, particularly as he continued to have recurring episodes of abscesses without repeated travel to endemic regions. It is possible that, while the patient may not have cleared his initial infection, his exogenous GHRH use may have contributed to the number of relapsing liver abscesses. Endogenous male hormones such as testosterone may contribute to these gender differences, as mice models show testosterone levels determine susceptibility to ALA.^14^ The relationship between GHRH and ALA has not been studied, but it may play a role similar to testosterone in increasing susceptibility.

## 4 CONCLUSION

We describe a case of recurrent ALA in a patient previously treated with metronidazole without repeated travel to an endemic area. Despite treatment with a 10‐day course of metronidazole 4 years ago for ALA, the patient developed three additional episodes of liver abscesses, ultimately found to be from amebiasis. This case highlights the importance of using appropriately dosed metronidazole, followed by an intraluminal agent, for effective treatment of ALAs. Imaging and serology are necessary for diagnosis, but PCR testing of stool and abscess fluid is critical. Drainage is not necessary but may be helpful in complicated ALAs.

## AUTHOR CONTRIBUTIONS

**Sasan D Noveir:** Writing – original draft; writing – review and editing. **Anh Hoang:** Writing – review and editing. **Katherine Li:** Writing – review and editing. **John C. Lam:** Supervision; writing – review and editing. **Khushboo Akkad:** Supervision; writing – review and editing.

## FUNDING INFORMATION

None.

## CONFLICT OF INTEREST STATEMENT

The author reports no conflicts of interest.

## CONSENT

Written informed consent was obtained from the patient to publish this report in accordance with the journal's patient consent policy.

## Data Availability

Data sharing not applicable to this article as no datasets were generated or analysed during the current study.
